# Efficacy of a telehealth cognitive behavioral therapy for improving sleep and nightmares in children aged 6–17

**DOI:** 10.3389/frsle.2024.1401023

**Published:** 2024-07-11

**Authors:** Lisa DeMarni Cromer, Sarah Beth Bell, Lauren E. Prince, Nicholas Hollman, Elissar El Sabbagh, Tara R. Buck

**Affiliations:** ^1^Department of Psychology, The University of Tulsa, Tulsa, OK, United States; ^2^Office for Research Development and Scholarly Activity, The University of Oklahoma School of Community Medicine, Tulsa, OK, United States; ^3^Department of Psychiatry, The University of Oklahoma School of Community Medicine, Tulsa, OK, United States

**Keywords:** nightmare treatment, insomnia child cognitive behavioral therapy, suicide risk, mental health treatment, child sleep

## Abstract

**Introduction:**

This study examined the efficacy of a five-module cognitive behavioral therapy for nightmares in children (CBT-NC) and improving sleep.

**Materials and methods:**

Forty-six youth aged 6–17 years with sleep problems and at least weekly chronic and distressing nightmares were randomized to treatment (*n* = 23) or waiting list (*n* = 23) using a block four randomized design. Among participants, 65% (*n* = 30) were White, 4% (*n* = 2) were Black/African American, 2% (*n* = 1) were Asian American, 13% (*n* = 6) were Native American or Pacific Islander, and 15% (*n* = 7) were multiracial. Fifty percent of participants (*n* = 23) were cisgender girls, 35% were cisgender boys (*n* = 16), 7% were transgender boys (*n* = 3), and 9% were gender non-binary (*n* = 4). The baseline nightmare persistence ranged from 6 months to 13.5 years. The treatment adapted exposure, relaxation, and rescription therapy for trauma-related nightmares in adults and added elements of cognitive behavioral therapy for insomnia in children. Psychoeducation included topics of sleep and nightmares, relaxation, anxiety management, and sleep hygiene; the youth were guided through nightmare exposure and rescription.

**Results:**

There was a statistically significant improvement in the number of nights with awakening (Cohen's *d* = 1.08), the number of weekly nightmares (Cohen's *d* = 0.82), and nightmare distress (Cohen's *d* = 1.05) for the treatment group compared to the wait-list group. Parent-reported youth sleep improved for the entire group from pretreatment to posttreatment (*p* < 0.001) but did not reach statistical significance for between-subjects analyses of the treatment group compared to the wait-list group (*p* = 0.05). Between-subjects analyses saw improvement for the treatment group compared to the wait-list group on internalizing and externalizing problems and suicidal thoughts and behaviors.

**Discussion:**

This study supports the efficacy of CBT-NC for improving sleep maintenance, nightmare frequency and distress, and other mental health difficulties in youth. Preliminary evidence of possibly improving suicidal thinking and behavior is also presented.

**Clinical trial registration:**

https://clinicaltrials.gov/study/NCT05588739, identifier: NCT05588739.

## Introduction

Chronic nightmares in children can cause a delay in sleep onset latency due to bedtime anxiety and can contribute to insomnia due to nighttime awakenings that disrupt sleep maintenance (Gieselmann et al., 2019). The presence of nightmares can increase the dropout of treatment for insomnia (Hamilton et al., 2023), suggesting that a combined nightmare and insomnia treatment could be ideal in cases of comorbid nightmare and insomnia disorders. Chronic nightmares are associated with myriad mental health and behavioral difficulties in youth (Gauchat et al., 2020), signal serious mental health problems (Gieselmann et al., 2019), and predict youth suicide even when controlling for other sleep problems (Liu et al., 2017). A systematic review of English-language studies found that 3%−6% of pediatric and 10%−12% of child psychiatric samples have a diagnosis of nightmare disorder (El Sabbagh et al., 2023). These estimates are likely low because nightmares are rarely included in routine clinical screening (Cromer et al., 2022b), and clinicians seldom diagnose nightmare problems as either stand-alone or co-occurring difficulties (Gieselmann et al., 2019).

Nightmares are often comorbid with insomnia, and patients with this comorbidity have worse mental health problems than those with insomnia alone (Paquet et al., 2024). Negative emotionality appears to explain the relationship between nightmares and mental health problems in youth (Nielsen, 2017; Gieselmann et al., 2019). Nightmares interrupt fear extinction and emotion regulation during rapid eye movement (Rousseau and Belleville, 2018). Nightmares are associated with daytime difficulties, including having a negative cognitive bias (Davis, 2009) and daytime distress and hyperarousal (Nielsen, 2017). Hyperarousal contributes to nightmare maintenance; therefore, experts recommend addressing hyperarousal in nightmare treatment (Gieselmann et al., 2019). Not surprisingly, fear extinction is believed to be central to nightmare treatments (Rousseau and Belleville, 2018). In examining mechanisms of change in nightmare treatments, two systematic reviews reported that improvements in self-efficacy were common among effective treatments (Rousseau and Belleville, 2018; Gill et al., 2023). The reviews reported that observed change factors were arousal, fear avoidance, and dysfunctional beliefs about sleep (Rousseau and Belleville, 2018).

Nielsen (2017) posited the stress-acceleration hypothesis (SAH) to explain the perniciousness of nightmares. Nielsen (2017) theorized that in the absence of criterion A trauma, the experience of early adversity alters brain architecture and creates a propensity for nightmares; SAH supports the idea of not distinguishing between idiopathic and posttraumatic nightmares. Although there is considerable longitudinal research to suggest that nightmares precede and predict later mental health difficulties (Gieselmann et al., 2019), SAH offers an alternative explanation for the relationship. SAH suggests that changes to brain architecture due to early adversity explain the relationship of nightmares to later mental health problems. To date, no experimental research has examined the nightmare–mental health relationship, and in a review, Lemyre et al. (2019) emphasized the need for research to examine the influence of nightmares on the development of primary mental health disorders. There is robust correlational and longitudinal research showing an association between nightmares and suicidality in youth (Liu et al., 2017; Stanley et al., 2017; Gieselmann et al., 2019; Gauchat et al., 2020; Kearns et al., 2020). Experimental research has not examined this relationship.

Cognitive behavioral therapy for insomnia is well-established for improving children's sleep onset latency, sleep efficiency (Lemyre et al., 2019), total sleep time, and wake after sleep onset (Blake et al., 2017; Gieselmann et al., 2019). Non-specific sleep or anxiety treatments may reduce nightmare frequency but not nightmare distress (Simard and Nielsen, 2009; Gieselmann et al., 2019). Because nightmare distress is most clearly related to mental health difficulties, nightmare treatment research is needed (Gieselmann et al., 2019); two systematic reviews have called for efficacy studies in youth (Gieselmann et al., 2019; Gill et al., 2023). Brief cognitive behavioral therapies for nightmares in children are promising, but sample sizes are small, studies are few (Gill et al., 2023), and efficacy studies have not been conducted (Lemyre et al., 2019). The existing studies point to imagery rehearsal and cognitive behavioral treatments as possibly effective (Gieselmann et al., 2019; Gill et al., 2023).

A promising treatment for nightmares in school-aged youth is cognitive behavioral therapy for nightmares in children (CBT-NC). Fernandez et al. (2013) initially developed the treatment for posttraumatic nightmares, adapting Davis's (2009) adult treatment for children. The treatment was later modified and manualized with a workbook for youth and a parent companion workbook that focused equally on sleep and posttrauma nightmares and incorporated some elements of cognitive behavioral therapy for insomnia (CBT-I) in children (Cromer et al., 2021). During pilot testing, children were sometimes confused by psychoeducation about trauma in Module 1 because they either had no trauma history or their trauma occurred during the infantile amnesia period. Given Nielsen's (2017) SAH theory of nightmares, the treatment was further modified to replace most of the trauma psychoeducation with additional CBT-I components. The modifications added sleep hygiene; bedtime routines (Mindell and Williamson, 2018); stress management, including a worry jar; relaxation components in each session (compared to two sessions in Fernandez's adaptation); positive imagery before bedtime; and mindfulness (Cromer et al., 2022a). Early evidence suggested that the treatment could be successful even when youth had co-occurring disorders, such as autism spectrum disorder and anxiety disorders (Cromer et al., 2023a), and a feasibility study found that children as young as 6 years old tolerated the treatment delivered over telehealth (Cromer et al., 2023b).

The current study sought to test the efficacy of the revised CBT-NC treatment manual. Because the study was initiated during the COVID-19 pandemic, it was conducted over telehealth. A 5-week wait-list control group was used as a comparison group for the five-module CBT-NC treatment group. Measures were collected from parents and youth at baseline, post-experimental condition, and, for the wait-list group, posttreatment. We predicted that the treatment group would improve compared to the wait-list group on outcomes examining sleep, nightmare frequency and distress, overall mental health, and suicidal ideation and behaviors. We also examined all variables using within-subjects analyses after the wait-list group completed treatment.

## Materials and methods

The University of Oklahoma Institutional Review Board approved the study. The study was registered with Clinical Trials (NCT05588739); data collection was from September 2020 to June 2023.

### Participants

Participants (*N* = 46) ranged in age from 6 to 17 years (*M* = 12.05 years, *SD* = 3.25 years). Table 1 presents participants' demographics and diagnoses. Sixty-five percent of participants (*n* = 30) were White, 4% (*n* =2) were Black/African American, 2% (*n* = 1) were Asian American, 13% (*n* = 6) were Native American or Pacific Islander, and 15% (*n* = 7) were multiracial. Among participants, 50% (*n* = 23) were cisgender girls, 34.7% (*n* = 16) were cisgender boys, 6.5% (*n* = 3) were transgender boys, and 8.7% (*n* = 4) were gender non-binary. Families' self-reported income ranged from $14,000 USD to $220,000 USD; the median range was $50,000–$74,599 USD. From parent report on the phone screen, most participants had at least one mental health diagnosis (*n* = 40, 87.0%), with a range from zero to five (*M* = 2.07, *SD* = 1.31) diagnoses. The modal diagnosis was anxiety disorder (*n* = 23, 50%). Nightmare persistence at baseline ranged from 6 months to 13.5 years. Trauma prevalence varied by reporter with 41 (89.1%) youth and 43 (93.5%) caregivers reporting that the child had at least one traumatic lifetime event; ranges were 0–13 (*M* = 4.22, *SD* = 3.18) events by youth report and 0–11 events according to caregivers (*M* = 3.63, *SD* = 2.43). Table 2 shows the prevalence of each type of reported trauma.

**Table 1:** Demographic characteristics between the treatment and the wait-list groups.

**Variable**	**Wait-list (*n* = 23) *M* (*SD*)**	**Treatment (*n* = 23) *M* (*SD*)**	**Full sample (*n=*46) *M* (*SD*) [mode]**
Age	11.4 (3.2)	12.7 (3.2)	12.05 (3.25) [9]
**Gender, % (** ***n*** **)**			
Cisgender girl	39.1 (9)	60.9 (14)	50.0 (23)
Cisgender boy	52.2 (12)	17.4 (4)	34.7 (16)
Transgender boy	4.3 (1)	8.7 (2)	6.5 (3)
Non-binary	4.3 (1)	13.0 (3)	8.7 (4)
**Race, % (** ***n*** **)**			
White or Caucasian	47.8 (11)	82.6 (19)	65.2 (30)
Black or African American	8.7 (2)	0.0 (0)	4.3 (2)
Asian American	0.0 (0)	4.3 (1)	2.2 (1)
Native American or Pacific Islander	21.7 (5)	4.3 (1)	13.0 (6)
Multiracial or other	21.7 (5)	8.7 (2)	15.2 (7)
**Ethnicity, % (** ***n*** **)**			
Hispanic or Latinx	13.0 (3)	0.0 (0)	6.5 (3)
Did not disclose	87.0 (20)	100.0 (23)	93.5 (43)
**Household income level, % (** ***n*** **)**			
Did not report	4.3 (1)	26.1 (6)	15.2 (7)
Under $15,000 USD	4.3 (1)	4.3 (1)	4.3 (2)
$15,000–24,999 USD	0 (0)	0.0 (0)	0.0 (0)
$25,000–34,999 USD	8.7 (2)	0 (0)	4.3 (2)
$35,000–49,999 USD	21.7 (5)	8.7 (2)	15.2 (7)
$50,000–74,999 USD	21.7 (5)	4.3 (1)	13.0 (6)
$75,000–99,999 USD	17.4 (4)	26.1 (6)	21.7 (10)
$100,000–149,999 USD	8.7 (2)	13.0 (3)	10.9 (5)
Greater than $150,000 USD	13.0 (3)	17.4 (4)	15.2 (7)
**Mental health disorders from phone screens, % (** ***n*** **)**			
Anxiety	39.1 (9)	60.9 (14)	50 (23)
Attention-deficit/ hyperactivity disorder	52.2 (12)	43.5 (10)	47.8 (22)
Depression	47.8 (11)	30.4 (7)	39.1 (18)
Posttraumatic stress disorder	26.1 (6)	11.7 (5)	23.9 (11)
Oppositional defiant disorder	13.0 (3)	8.7 (2)	10.9 (5)
Bipolar disorder	0 (0)	13.0 (3)	6.5 (3)
Autism spectrum disorder	4.3 (1)	4.3 (1)	4.3 (2)
Disruptive mood dysregulation disorder	8.7 (2)	0 (0)	4.3 (2)
Insomnia disorder	4.3 (1)	4.3 (1)	4.3 (2)
Obsessive-compulsive disorder	4.3 (1)	4.3 (1)	4.3 (2)
Adjustment disorder	4.3 (1)	0 (0)	2.2 (1)
Attachment disorder	4.3 (1)	0 (0)	2.2 (1)
Conversion disorder	0 (0)	4.3 (1)	2.2 (1)
Nightmare disorder	0 (0)	4.3 (1)	2.2 (1)
Sensory processing	4.3 (1)	0 (0)	2.2 (1)

**Table 2:** Youth lifetime traumatic experiences from the child and adolescent trauma screen at baseline.

**Traumatic experiences**	**Caregiver report % (*n*)**	**Child report % (*n*)**
Serious natural disaster	32.6 (15)	28.3 (13)
Serious accident or injury	39.1 (18)	52.2 (24)
Robbed by threat, force, or weapon	2.2 (1)	4.3 (2)
Slapped, punched, or beat up in the family	19.6 (9)	39.1 (18)
Slapped, punched, or beat up by somebody not in the family	26.1 (12)	34.8 (16)
Seeing someone in the family get slapped, punched, or beat up	32.6 (15)	28.3 (13)
Seeing someone in the community get slapped, punched, or beat up	8.7 (4)	21.7 (1)
Someone older touching their private parts	23.9 (11)	28.3 (13)
Someone forcing or pressuring sex or when they could not say no	17.4 (8)	23.1 (12)
Someone close to the child dying suddenly or violently	37.0 (17)	39.1 (18)
Attacked, stabbed, shot at, or hurt badly	6.5 (3)	10.9 (5)
Seeing someone attacked, stabbed, shot at, hurt badly, or killed	10.9 (5)	21.7 (10)
Stressful or scary medical procedure	39.1 (18)	34.8 (16)
Being around war	0 (0)	0 (0)
Any other event	57.4 (31)	52.2 (24)

### Recruitment

The study was advertised on Facebook and with flyers at community clinics, which collectively yielded six participants. Additionally, clinicians at the university made referrals (*n* = 8), and we recruited participants by telephone solicitation from a patient pool at the university's Child Psychiatry Clinic (*n* = 32). If parents reported that their child had weekly nightmares and met other inclusion criteria, they were invited to complete a phone screen to confirm eligibility. Inclusion criteria were being ages 6–17 years, experiencing weekly nightmares that caused awakening, the youth reading English at a first-grade level (by parent report), living in Oklahoma, and medications being stable for 1 month. Untreated sleep apnea and currently being in a mental health crisis (e.g., child being in an inpatient facility) were exclusion criteria. Participants who were not eligible were told to reach out to the study phone number if anything changed.

**Figure 1:**
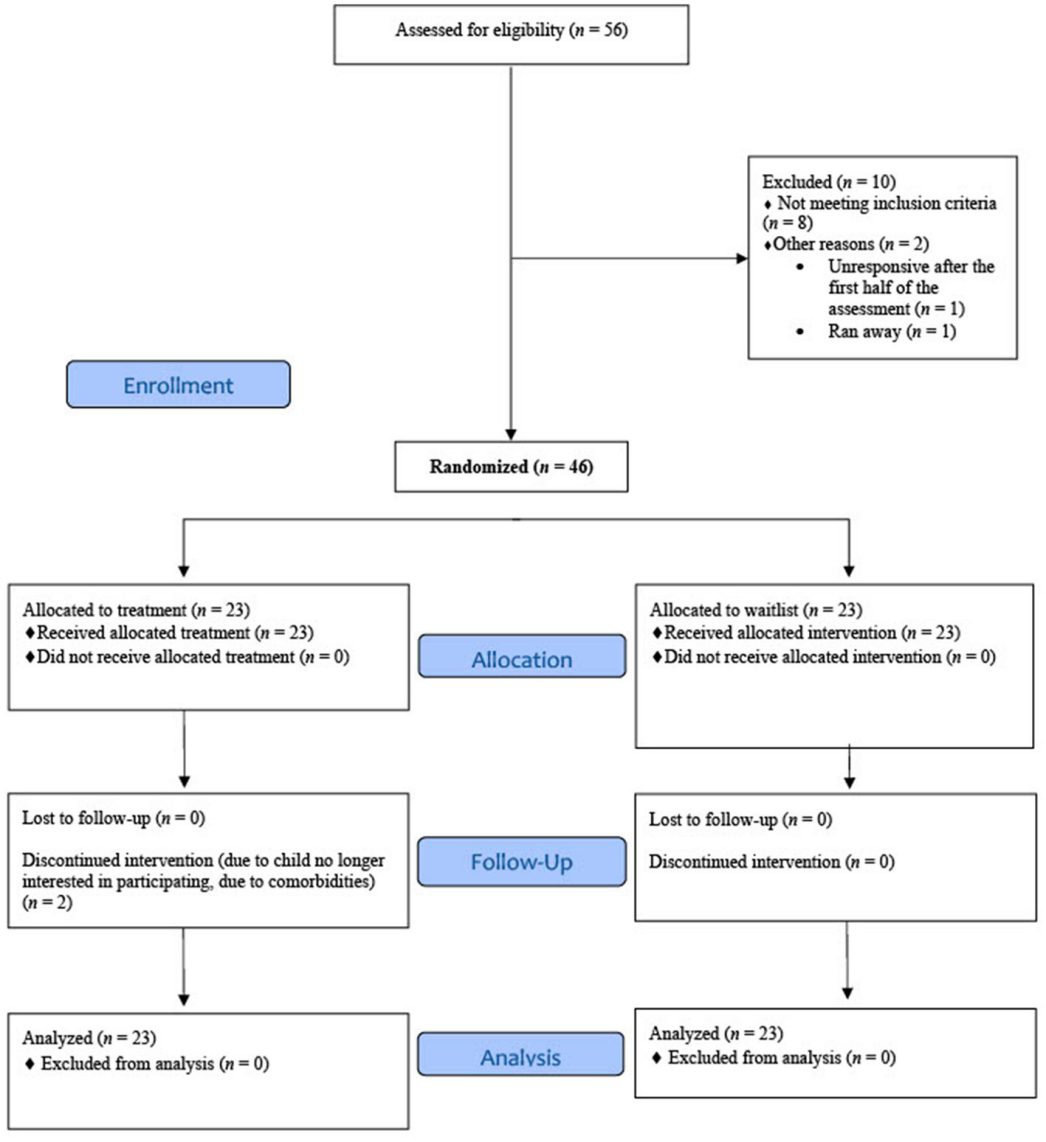
Consort diagram.

A total of 1,298 recruitment calls and inquiries were made. Of these, 849 (65.4%) were ineligible to phone screen because they did not report weekly nightmares or they did not meet other inclusion criteria, 334 (25.7%) were unresponsive, 25 (1.9%) declined the phone screen, and 90 (6.9%) were eligible and completed a phone screen. From the phone screens, 14 (15.6%) were ineligible, 12 (13.3%) were eligible but declined participation, and 64 (71.1%) were eligible and attended an orientation. Of those who attended a study orientation, 58 (90.6%) consented to participate. Two people (3.4%) were unresponsive after consent. Of those who completed the baseline assessment, 8 (13.8%) were excluded because their nightmares did not meet frequency criteria; 1 (1.7%) was unresponsive; 1 (1.7%) ran away from home prior to randomization and, subsequently, the child could not be reached. The remaining 46 (79.3%) were eligible and randomized either to the treatment (*n* = 23; 50%) or wait-list (*n* = 23; 50%) group (Figure 1) using a block-four randomized design.

### Procedure

The study orientation (*n* = 64) with the child and parent occurred over Zoom. We explained the study using a short graphic novel, photos of the treatment team, and a study timeline. We emphasized time expectations and the importance of completing the study to the end. Consent was offered as a separate appointment; however, to be flexible, if a family wanted to consent immediately, this was permitted. Forty-one (70.7%) consented immediately, and 17 (29.3%) delayed. Following consent, a baseline assessment was scheduled to verify eligibility. The randomization assignment was a separate phone call following baseline.

Treatment started the week following randomization; those on the waiting list were assessed after 5 weeks and then invited to treatment. All sessions were on a health care Zoom platform. Families received a $10 USD Amazon gift card for the consent, baseline assessment, and each therapy appointment. For each post-assessment, they received a $30 USD gift card. In total, the treatment group received $100 USD, and the wait-list group received $130 USD. Families were informed about the gift cards during the phone screen as a participation incentive. Additionally, the child selected gifts valued at approximately $5 USD from a virtual treasure box at the conclusion of each appointment; they were permitted to defer gifts each week to accumulate larger amounts for more desirable gifts. Most participants deferred gifts at least once during the study for the modal treasure box item being about $10 USD (*n* = 39, 84.8%).

Youth were assessed on sleep, nightmares, trauma history, and suicidal thoughts and behaviors (STBs). Parents completed the measures about youth mental health and trauma history. Trauma history was collected to understand whether all children with chronic nightmares experienced trauma. For youth who endorsed STBs, the therapist or assessor conducted a suicide risk assessment with the participant and family and determined the appropriate plans of action; this was the case for youth in both experimental conditions. Suicide prevention followed standard suicide risk assessment practices and ranged from coordinating with a community mental health provider, implementing a safety plan, or providing a referral for acute crisis mental health treatment. Parents were brought into session to support the safety plan. The team was advised of safety plans through a continuity-of-care file so that all members could follow up on STB concerns.

### Measures

#### Sleep and nightmare diaries

Daily sleep and nightmare diaries were used to record the previous night's number of awakenings from nightmares and the level of distress. Distress was queried on a thermometer that had an emoji face scale from *happy* (1) to *very stressed* (10). The prompt was “*How upsetting were they?*”

#### Child behavior checklist for ages 6–18 parent report

The Child Behavior Checklist for Ages 6–18 (CBCL) parent report is a standardized machine-scored instrument that provides ratings for 20 competence and 120 problem items (Achenbach and Rescorla, 2001). Scales yield Internalizing, Externalizing, and Total Problems *T*-scores. *T*-scores between 60 and 63 are borderline clinical, and scores >64 are clinically significant (Achenbach and Rescorla, 2001). Responses are how “each item describes your child now or within the last 6 months.” The CBCL has six diagnostic scales: Affective Problems, Anxiety Problems, Somatic Problems, Attention Deficit/Hyperactivity Problems, Oppositional Defiant Problems, and Conduct Problems for which scores between 64 and 69 are borderline clinical, and scores >70 are considered clinically significant problems (Achenbach and Rescorla, 2001). The CBCL internal consistency for the subscales' Cronbach's alphas ranged from 0.78 to 0.97, and they had strong convergent validity with the Behavior Assessment System for Children (*r* = 0.85–0.89; Achenbach and Rescorla, 2001).

CBCL Items 76 (“sleeps less than most kids”) and 100 (“trouble sleeping”) have been used to index insomnia problems (Mancini and Pearcy, 2021). Mancini and Pearcy (2021) conducted an exploratory factor analysis of seven sleep-related items on the CBCL and found that Items 76 and 100 were correlated with each other (Cronbach's alpha = 0.78) and with the Sleep Disorders Scale for Children subscale for initiating and maintaining sleep (*r* = 0.57–0.70). Based on Mancini and Pearcy (2021), we conducted the analyses with these two items with a possible summed score from 0 to 4. At baseline, Cronbach's alpha was 0.69, and at post-condition, Cronbach's alpha was 0.51.

#### STBs

Eight questions queried yes/no for lifetime and last week suicidal ideations, plans, and attempts. Youth were asked the questions based on Wong et al. (2011), Stanley et al. (2017), and Russell et al. (2018).

#### The child and adolescent trauma screen

The Child and Adolescent Trauma Screen (CATS) is a 15-item checklist of lifetime traumatic events (Sachser et al., 2017). If any trauma is endorsed, participants are asked 20 posttraumatic stress symptom questions regarding the most upsetting trauma. Symptoms are reported based on frequency over the last 2 weeks from 0, *never*, to 3, *almost always*. The total score is summed with a possible range from 0 to 60. Scores of 21 or higher indicate clinically relevant symptoms (Sachser et al., 2017). The CATS has shown good to excellent reliability (α = 0.88–0.94) and medium to strong convergent validity (*r* = 0.40–0.82; Sachser et al., 2017). In the current study, the CATS was administered via interview with youth and parents separately (i.e., administered twice).

#### Treatment

The CBT-NC (Cromer et al., 2019) provides a manualized workbook with educational material and activities for children; there is a companion parent workbook. See Table 3 for a description of the content and activities for all five modules. Modules 1–3 address sleep problems. Module 4 addresses nightmares through exposure and rescription, and Module 5 cultivates sleep and dream efficacy and maintenance planning. The modules are designed to be progressive and be completed in a single session. If a module is not mastered, for example, a sleep routine was not implemented, and the content is reviewed before moving on to the next module.

**Table 3:** CBT-NC treatment components.

**Module**	**Psychoeducation topics**	**Goals**	**Activities**
1	Orientation to CBT and treatment; understand the value of good sleep and of sleep routines	Instill hope and increase motivation for change; develop relaxation skills; cultivate sleep efficacy; teach relaxation through breathing techniques	Drawing of life with no nightmares; use of “sleep deprivation goggles” for educational demonstration; thought/feeling/behavioral spiral following a nightmare; develop or enhance existing bedtime routine; belly breathing
2	Sleep stages and benefits of sleep; the role of avoidance in stress maintenance; sleep hygiene	Increase sleep efficacy; modify any poor sleep hygiene; reduce or manage worrying in the evening with a worry jar/box	Creation and use of worry box; modifications to sleep routine developed previous week
3	Brains are malleable and can be taught that beds are for sleeping; physiological reactions to stress and how to change them	Increase sleep and dream efficacy by imagining pleasant things before bed; progressive muscle relaxation; continue to enhance/shape sleep hygiene	Draw pleasant words or images on a pillowcase; progressive muscle relaxation; modify sleep routine if needed, and enhance sleeping environment
4	Development and maintenance of nightmares; rationale for exposure treatment	Exposure to trauma nightmare, develop nightmare change efficacy by preparing for exposure; rescript the nightmare and practice rescription; develop new relaxation skill	Identify a time when they overcame a fear; write out or draw the nightmare; rescript the nightmare based on a salient theme; read the new nightmare and modify until it is liked; practice slow breathing
5	Relapse prevention and maintenance planning	Review of treatment and cultivate efficacy for skills learned; plan for relapse prevention including rescripting any future nightmares	Review of four modules and fill a virtual toolbox with skills they found helpful and identify situations when to use them; plan for rescripting future nightmares that could occur

CBT-NC, cognitive behavioral therapy for nightmares in children; CBT, cognitive behavioral therapy.

### Statistical analysis

All analyses were conducted using SAS v9.4 with a significance level of α = 0.05. Two outliers (>3 *SD* above/below the mean) were found for the total number of nightmares in the past week. All analyses were conducted with and without the outliers; because outliers did not significantly alter the results, we present all analyses with them included. There was some missing sleep tracker data due to experimenter error. If there were no sleep tracker data, the children's answers to the question, “How many nights in the past week have you had a nightmare?” were used for the number of nights with nightmares (*n* = 11). Where data was missing, the mean imputation was used for baseline, and the last observation carried forward was used for missing data after baseline in line with the intention-to-treat design. To test for significant differences between the wait-list and treatment groups, independent samples *t*-tests were used to evaluate Hypothesis 1, and a Wilcoxon rank-sum test was used to evaluate the ordinal data for Hypothesis 2. When variances for the treatment and wait-list groups significantly differed, the Satterthwaite method was used to approximate degrees of freedom in the independent samples *t*-test. For within-group exploratory analysis testing for significant differences between baseline and posttreatment for the entire sample that had treatment regardless of assigned condition, paired sample *t*-tests were used for Hypothesis 1, and a Wilcoxon signed-rank test was used for Hypothesis 2. There were three measurements/data points across the study: baseline, post-condition (following treatment or following 5 weeks on the waiting list), and a third time point for the wait-list group only, following their treatment. For within-group exploratory analysis on the entire sample, the post-condition value was used for the wait-list group's baseline so we could test the effect on the treatment using a uniform baseline measure for the entire sample right before they were treated. Two participants stopped treatment after Module 2, but they completed post-condition assessments. They were included in all analyses as intent to treat. Reliable change indexes (RCI) were calculated for CBCL subscale scores using the formula suggested by Christensen and Mendoza (1986) to test for meaningful differences between baseline and post-condition. This formula required data from a normative sample; Achenbach and Rescorla (2004) study was used. A reliable change index of greater than 1.96 is considered to be a reliable change (Christensen and Mendoza, 1986).

## Results

**Figure 2:**
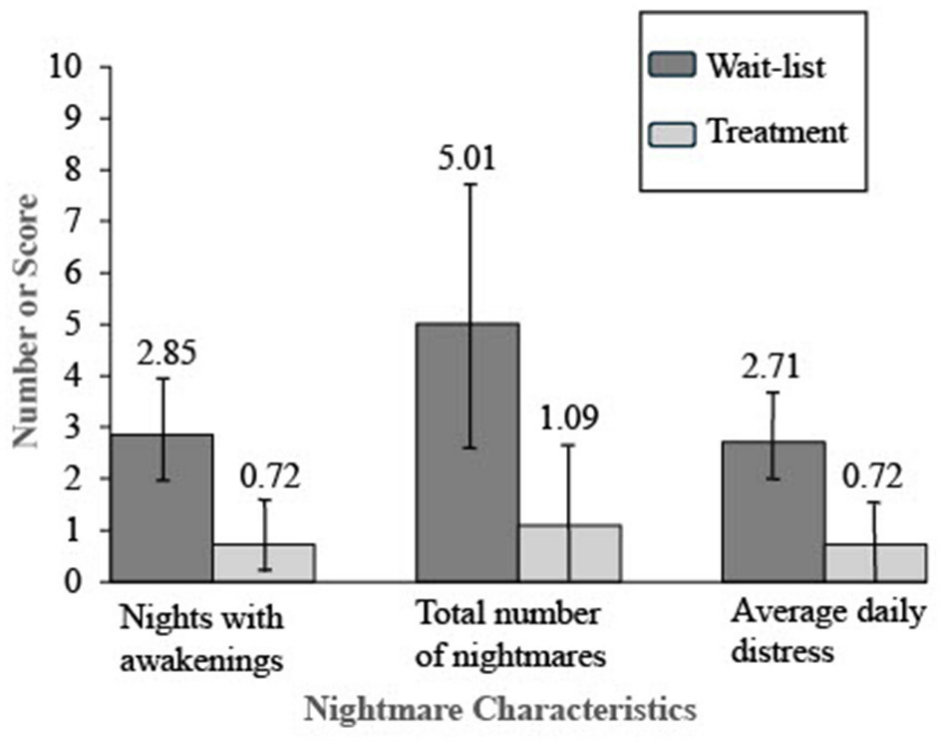
Nightmare characteristics in the last week following treatment or waitlist.

Hypothesis 1 predicted the treatment group would have better outcomes than those in the wait-list group on child-reported sleep maintenance as well as nightmare frequency and distress. There were no statistically significant between-group differences in outcome variables at baseline. Following the experimental condition, those in the treatment group had better outcomes on all three measures compared to those in the wait-list group. They had statistically significant improvements in the number of nights with awakenings indexing sleep maintenance, nightmare frequency, and nightmare distress. Table 4 presents the means, standard deviations, independent samples *t*-test values, and Cohen's *d* effect sizes for all three outcome variables and reports between-group differences. Figure 2 displays the means and 95% confidence intervals for these results following treatment.

**Table 4:** Nightmare characteristics in the last week following treatment or wait-list.

	**Wait-list *M* (*SD*)**	**Treatment *M* (*SD*)**	**Difference**	**Confidence interval**	***t*-score**	**df**	***p*-value**	**Cohen's *d***
Nights with awakenings	2.85 (2.31)	0.72 (1.57)	2.13	0.96, 3.31	3.65	44.0	< 0.001	1.08
Total number of nightmares	5.01 (5.98)	1.09 (3.13)	3.93	1.09, 6.76	2.79	33.22	0.009	0.82
Average daily distress	2.71 (2.01)	0.72 (1.78)	1.99	0.86, 3.12	3.56	44.0	< 0.001	1.05

Hypothesis 2 predicted that the treatment group would have better outcomes than the wait-list group on the parent-reported CBCL sleep composite variable. The treatment and wait-list groups' median values did not significantly differ at baseline on the parent-reported CBCL sleep composite variable (*p* = 0.305); the wait-list group median was 3.00 [interquartile range (*IQR*): 2.00–4.00], and the treatment group median was 2.00 (*IQR*: 1.00–4.00). Following treatment, there was no significant difference between the groups on the CBCL sleep composite variable (*p* = 0.053), although the treatment group had a lower median of 1.00 (*IQR:* 1.00–2.00) after treatment compared to the wait-list control median of 2.00 (*IQR*: 2.00–4.00) at the same time point.

**Figure 3:**
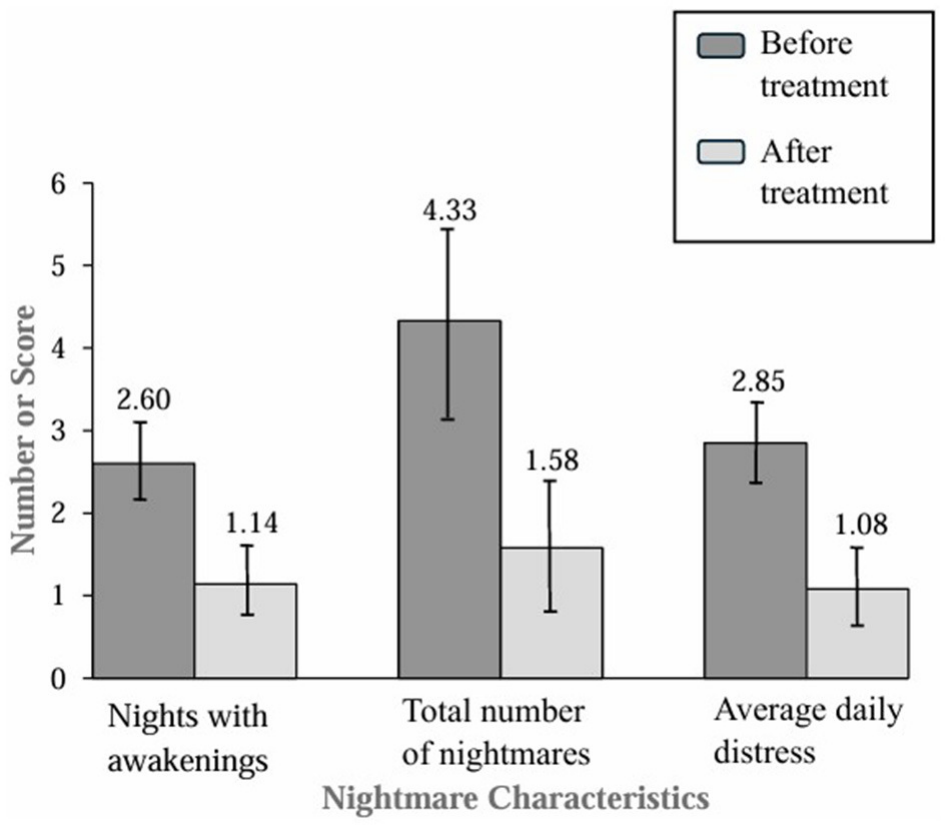
Nightmare characteristics in the last week in the combined sample before and after treatment.

Exploratory analyses testing for within-group differences from baseline to posttreatment for the entire sample were conducted after the initial analyses testing for between-group differences. After the entire sample had been treated, there were statistically significant improvements in sleep maintenance, nightmare frequency, and nightmare distress. The combined sample had an average decline of 1.46 [95% CI (0.92, 1.99)] nights in the past week with awakenings compared to before treatment, dependent samples *t*_(45)_ = 5.48, *p* < 0.001, Cohen's *d* = 0.81. Additionally, there were 2.75 [95% CI (1.42, 4.08)] fewer nightmares reported in the past week compared to before treatment, dependent samples *t*_(45)_ = 4.15, *p* < 0.001, Cohen's *d* = 0.61. The combined sample average nightmare distress was 1.77 [95% CI (1.09, 2.45)] points lower than before treatment, dependent samples *t*_(45)_ = 5.27, *p* < 0.001, Cohen's *d* = 0.78. Figure 3 displays the within-group results graphically.

For the exploratory analyses' results testing for within-group differences on the CBCL sleep composite variable, a Wilcoxon sign-rank test indicated that median values after treatment for the CBCL sleep composite variable were significantly lower than before treatment (*S* = 188.0, *p* < 0.001). The median CBCL composite score for the complete sample was 2.00 (*IQR*: 1.00–4.00) before treatment and 1.00 (*IQR*: 0.00–2.00) after treatment.

Hypothesis 3 predicted that the treatment group would have better outcomes than the wait-list group on parent-reported youth mental health and child-reported STB. In examining the RCIs for the CBCL for the wait-list group, only the Attention Problems subscale (*n* = 6, 26.1%) evidenced reliable change from baseline to postcondition, meaning that they did not improve in other indices of mental health. In contrast, other than the School CBCL subscale, there was reliable change observed in every other CBCL subscale for the treatment group from baseline to posttreatment, meaning that the treatment group saw significant improvements in mental health. The highest prevalence of reliable change improvement was for the Anxiety/Depression (*n* = 7, 30.4%), Attention Problems (*n* = 8, 34.8%), and Obsessive Compulsive (*n* = 7, 30.4%) subscales. No reliable change was found for the internalizing, externalizing, or total problems scales for the wait-list group. In contrast, for the treatment group, reliable changes were found for six (26.1%) participants on the Internalizing Problems scale, five (21.7%) participants on the Externalizing Problems scale, and four participants (17.4%) on the Total Problems Scale.

At baseline, 31 (67.39 %) participants reported lifetime history of STBs; five (21.7%) youth in the treatment group and five youth (21.7%) in the wait-list group reported STBs in the week prior to the baseline assessment. After the experimental condition, only one youth (4.3%) in the treatment group and two (8.6%) in the wait-list group, who reported STB at baseline, continued to endorse recent STB postcondition. Additionally, two (8.6%) in the wait-list group who did not report STB at baseline reported STB in the previous week at their postcondition assessment compared to no new individuals in the treatment group.

## Discussion

This study evaluated the efficacy of CBT-NC for improving sleep and nightmares in pediatric samples. There is growing evidence of the value of CBT-I in children (Ma et al., 2018). However, there is also evidence that CBT-I alone does not improve nightmares, and there is a pressing need for nightmare-specific treatment research in pediatric populations (Gieselmann et al., 2019; Gill et al., 2023). The current study examined the effects of a cognitive behavioral treatment that included elements of insomnia and nightmare-focused treatments, on the proximal outcomes for sleep and nightmares, and whether there were generalized benefits to other mental health problems. The treatment targeted sleep and nightmares, regardless of nightmare etiology. The treatment adaptations for CBT-NC were informed by Nielsen's (2017) stress acceleration hypothesis of nightmares and incorporated elements of CBT-I as well as relaxation and mindfulness to target mechanisms that are hypothesized to maintain nightmares. The skills in the treatment related to several mechanisms of change included reducing hyperarousal (Nielsen, 2017; Gieselmann et al., 2019), improving self-efficacy (Rousseau and Belleville, 2018; Gill et al., 2023), and reducing negative emotionality (Nielsen, 2017; Gieselmann et al., 2019), daytime distress (Nielsen, 2017), and fear extinction (Rousseau and Belleville, 2018). Treatment occurred over five progressive modules, starting with psychoeducation in the first three modules, exposure and rescription in the fourth module, and relaxation, imagery, and maintenance planning in the fifth module.

We evaluated the treatment's impact on sleep maintenance, nightmare frequency, nightmare distress, sleep-related difficulties, and overall mental health. Previous correlational research with youth suggested that chronic nightmares and nightmare distress are related to mental health difficulties (Lemyre et al., 2019; Gauchat et al., 2020). We hypothesized that the treatment group would have better outcomes than the wait-list group on nightmare frequency and distress, sleep problems, and overall mental health, including STBs.

Results support the efficacy of CBT-NC for improving sleep maintenance, nightmare frequency, and distress in youth aged 6–17 with and without a trauma history. Compared to those in the wait-list group, participants in the treatment group had fewer statistically significant nights with awakenings, a lower overall nightmare frequency, and less nightmare distress following treatment. These findings provide evidence for the efficacy of CBT-NC for nightmare frequency and distress. Consistent with small-*N* studies with youth, and promising other research in adults (Gieselmann et al., 2019; Gill et al., 2023), cognitive behavioral techniques practiced over a brief period (about 5 weeks), can be effective for improving sleep maintenance and reducing nightmares in youth.

The study also explored the impact of CBT-NC on overall sleep quality using the parent report of sleep on the CBCL. There was not a statistically significant difference in parent-reported youth sleep following the experimental condition (*p* = 0.053). Within-subjects comparisons after everyone had been treated found that from pretreatment to posttreatment, there was a statistically significant improvement in sleep on the CBCL sleep items. The lack of a statistically significant difference between groups following the 5 weeks of experimental condition could be due to not having enough statistical power, or measurement sensitivity, especially given that parents are not always aware of their child's sleep. Additionally, the CBCL queried “now or in the past 6 months,” which may have lacked sensitivity to changes in sleep over the prior 5 weeks of treatment.

Exploratory data analysis examined whether the treatment group would have better outcomes than the wait-list group on parent-reported youth mental health and youth self-reported STB. Following completion of CBT-NC, the treatment group showed improvements across nearly all CBCL subscales, including Anxiety, Depression, Attention Problems, and Obsessive Compulsive symptoms, whereas the wait-list group remained unchanged on these subscales. The benefit possibly was due to the general mental health benefits associated with cognitive behavioral therapies. There is robust research to show that relaxation techniques and psychoeducation that promote self-efficacy can improve mental health (Wergeland et al., 2021). Consistent with theories of nightmare maintenance (Rousseau and Belleville, 2018), CBT-NC likely disrupts the nightmare cycle by reducing nightmare distress, improving self-efficacy, and reducing daytime hyperarousal as nightmares decline, thereby accounting for the observed improvements in overall mental health.

We also examined STBs at baseline and following treatment; all who endorsed anything more than passive ideation had safety plans conducted with their assessor or therapist at the time of the STB disclosure. Five youth in each conditioned endorsed STBs at baseline. Following their respective experimental conditions, one youth in the treatment group and four in the wait-list group endorsed STBs. Interestingly, two participants on the waiting list and none in the treatment condition developed new STBs following the experimental condition, suggesting that while improvements in STBs may be due to the safety planning, a benefit of the nightmare treatment may be preventing new STBs. This is a small sample and therefore should be interpreted tentatively. Nonetheless, given that nightmares are theorized to be an alarm signal for suicidality (Liu et al., 2017; Kearns et al., 2020), efforts to remove nightmares and track possible reductions in STBs could be an ethical experimental paradigm for understanding the connection.

### Implications

The results of this study have several implications. Gill et al. (2023) stated there is an urgent need for efficacy studies on nightmare treatment in youth. The current study answers this call, and it provides evidence of the treatment efficacy of CBT-NC via telehealth. The success of using telehealth increases inclusivity for those with barriers to in-person treatment. Addressing chronic nightmares may offer a pathway to improving overall youth mental health, possibly by mitigating symptoms associated with other psychiatric disorders. Many participants improved on mental health indices on the CBCL, supporting the nightmare–mental health connection. Also, the lower levels of STBs in the treatment group compared to those on the wait-list suggest that additional exploration into the nightmare–suicide contiguity is a worthwhile line of research. Given that the sample was diverse and included Native American as well as transgender and gender non-binary youth, all being groups that have far higher rates of suicidality than their peers, this research also has the potential for reducing health inequities by improving sleep and reducing STBs in these high-risk groups.

### Limitations

There are measurement limitations to the current study. Actigraphy or other objective sleep measurements were not used, and we only had the CBCL to look at sleep-related concerns. Additionally, mental health diagnoses were obtained from parent report rather than from a file review, and we did not query whether youth had current or past unprescribed substance use. The sample was recruited from an outpatient psychiatric clinic, and the results may not generalize to other populations. There is potential sample bias in recruiting via telephone because not everyone responds to telephone solicitation. The small sample size and low power prevented the use of path analysis or analyses of covariance, which would control for covariates.

The nightmare research in youth is nascent, and we are not aware of research that sufficiently guides how to quantify how much of a change in nightmare frequency or distress is clinically meaningful. A corollary of the measurement issues with nightmares is determining whether there is a meaningful nightmare–suicide contiguity. Furthermore, the small samples for youth with STBs, while interesting, are too small to consider to be promising; nonetheless, it justifies further investigation.

### Future directions

Although CBT-NC is a relatively brief treatment, participating in a treatment study is burdensome for families. Baseline and follow-up assessments that are required for meaningful research data add a burden. Although our team previously conducted pilot studies to refine research methodology to increase retention (Cromer et al., 2023b), the data collection was slow due to budget constraints that limited personnel. The current study represents 3 years of data collection. Future research with more resources will allow for higher enrollments. Additionally, future research will benefit from an ecological momentary assessment to reduce missing data; additionally, the methodology could be improved with objective sleep measures and measures that are sensitive to change. Confidence in the efficacy of CBT-NC can be achieved with other research teams conducting replication research, and from following up with participants over longer periods to evaluate whether nightmare improvements remain. There are myriad reasons why nightmares and suicide could be related, for example, a lack of self-efficacy (Rousseau and Belleville, 2018; Gill et al., 2023), low executive function and high impulsivity (Gauchat et al., 2020), or general daytime distress (Nielsen, 2017). Future research with larger samples should examine mediators of change.

### Conclusion

Youth with chronic nightmares appear to benefit from this five-module cognitive behavioral treatment with reductions in nighttime awakenings, nightmare frequency, and distress. Additionally, the treatment is promising for possible secondary benefits to mental health. The treatment is tolerated as seen by high retention rates and, like adult treatments for nightmares (Gill et al., 2023), may well be a first-line treatment for chronic nightmare sufferers, providing relief after only 5 weeks of treatment.

## Data availability statement

The raw data supporting the conclusions of this article will be made available by the authors, without undue reservation.

## Ethics statement

The studies involving humans were approved by The University of Oklahoma Health Science Center Institutional Review Board. The studies were conducted in accordance with the local legislation and institutional requirements. Written informed consent for participation in this study was provided by the participants' legal guardians/next of kin.

## Author contributions

LC: Conceptualization, Funding acquisition, Methodology, Project administration, Supervision, Visualization, Writing – original draft, Writing – review & editing. SB: Formal analysis, Methodology, Writing – review & editing. LP: Data curation, Project administration, Validation, Visualization, Writing – original draft, Writing – review & editing. NH: Formal analysis, Visualization, Writing – original draft, Writing – review & editing. EE: Data curation, Project administration, Writing – original draft. TB: Conceptualization, Funding acquisition, Methodology, Project administration, Resources, Supervision, Writing – review & editing.
